# Computed tomographic detection of a spontaneous cervical spinal epidural hematoma in a patient without overt neurological deficits

**DOI:** 10.1093/omcr/omag085

**Published:** 2026-06-08

**Authors:** Tomoya Yamaguchi, Hikari Matsumura, Yusuke Hiraba, Moe Sekiguchi, Naoya Ishibashi, Sayaka Kinugasa, Koshiro Takano, Hajime Shishido, Kenya Kawakita

**Affiliations:** Emergency Medical Center, Kagawa University Hospital, 1750-1 Ikenobe, Miki, Kita-gun, Kagawa 761-0793, Japan; Emergency Medical Center, Kagawa University Hospital, 1750-1 Ikenobe, Miki, Kita-gun, Kagawa 761-0793, Japan; Emergency Medical Center, Kagawa University Hospital, 1750-1 Ikenobe, Miki, Kita-gun, Kagawa 761-0793, Japan; Emergency Medical Center, Kagawa University Hospital, 1750-1 Ikenobe, Miki, Kita-gun, Kagawa 761-0793, Japan; Emergency Medical Center, Kagawa University Hospital, 1750-1 Ikenobe, Miki, Kita-gun, Kagawa 761-0793, Japan; Emergency Medical Center, Kagawa University Hospital, 1750-1 Ikenobe, Miki, Kita-gun, Kagawa 761-0793, Japan; Emergency Medical Center, Kagawa University Hospital, 1750-1 Ikenobe, Miki, Kita-gun, Kagawa 761-0793, Japan; Emergency Medical Center, Kagawa University Hospital, 1750-1 Ikenobe, Miki, Kita-gun, Kagawa 761-0793, Japan; Emergency Medical Center, Kagawa University Hospital, 1750-1 Ikenobe, Miki, Kita-gun, Kagawa 761-0793, Japan; Department of Emergency, Disaster, and Critical Care Medicine, Faculty of Medicine, Kagawa University, 1750-1 Ikenobe, Miki, Kita-gun, Kagawa 761-0793, Japan

**Keywords:** spontaneous spinal epidural hematoma, cervical spine, acute spinal pain, conservative management, computed tomography

## Abstract

Spontaneous spinal epidural hematoma (SSEH), with an annual incidence of 0.1 per 100 000 individuals, typically presents with acute spinal pain followed by neurological deficits. We describe the case of a 73-year-old woman who presented with acute posterior neck and back pain without overt neurological deficits. While contrast-enhanced computed tomography (CT) was primarily performed to exclude aortic dissection, it played a critical role in incidentally identifying a hyperattenuating epidural lesion, which was subsequently confirmed as a dorsal cervical epidural hematoma by magnetic resonance imaging. Given the patient’s stable neurological condition, conservative management was adopted, resulting in complete recovery without sequelae. This case underscores that SSEH may manifest solely as pain and that CT, even when performed to exclude other vascular emergencies, can provide an incidental yet critical initial diagnostic clue, facilitating timely diagnosis and appropriate management.

## Introduction

Spontaneous spinal epidural hematoma (SSEH) typically presents with acute spinal pain and progressive neurological impairment, requiring prompt recognition to prevent permanent deficits [1, 2]. While magnetic resonance imaging (MRI) remains the diagnostic gold standard [2–4], computed tomography (CT) performed for other vascular emergencies can provide the first diagnostic clue if images are meticulously evaluated [5]. Here, we describe a patient presenting solely with pain in whom cervical SSEH was initially suspected based on CT findings, illustrating the importance of recognizing this entity in emergency settings, even without overt neurological deficits.

## Case report

A 73-year-old woman presented to the emergency department with the sudden onset of posterior neck and back pain. She had no history of trauma or hypertension and was not taking anticoagulant or antiplatelet agents. On arrival, her vital signs were stable. While the patient initially reported mild and transient paresthesia in the right upper limb involving the C6 and C7 dermatomes, this symptom resolved spontaneously upon arrival. A subsequent thorough neurological examination revealed no objective motor weakness, persistent sensory loss, or sphincter dysfunction. Consequently, the patient was considered to have no overt neurological deficits at the time of initial evaluation. Laboratory investigations, including platelet counts and coagulation studies, were within normal limits. The acute onset of pain necessitated the exclusion of life-threatening conditions such as acute aortic dissection. Contrast-enhanced CT, evaluated using soft-tissue windows (level 40 HU, width 350 HU), revealed no vascular abnormalities but demonstrated a hyperattenuating lesion in the epidural space surrounding the cervical and upper thoracic spinal cord on axial images (Fig. 1). Subsequent MRI, performed immediately after CT, confirmed a predominantly dorsal cervical epidural hematoma extending from C4 to T2, consistent with SSEH (Fig. 2A). Due to her stable status after symptom resolution, conservative management was initiated, including analgesia, blood pressure control, and close neurological monitoring. A follow-up MRI on day 4 after admission demonstrated a reduction in hematoma size (Fig. 2B). The patient was discharged on day 5 without neurological sequelae and remained asymptomatic, with no recurrence at the 6-month follow-up.

**Figure 1:**
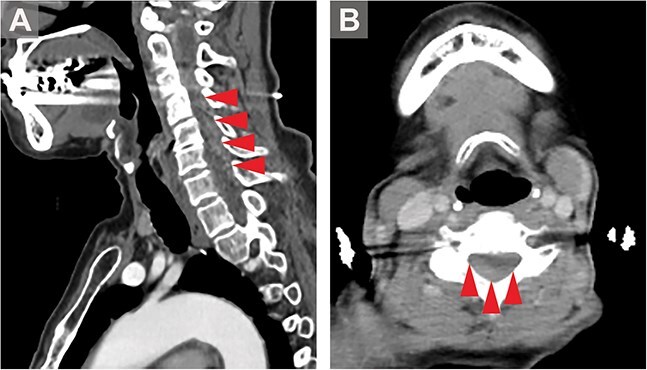
Contrast-enhanced CT on admission demonstrates a hyperattenuating lesion in the posterior epidural space on sagittal (A) and axial (B) views (arrowheads), consistent with epidural hematoma.

**Figure 2:**
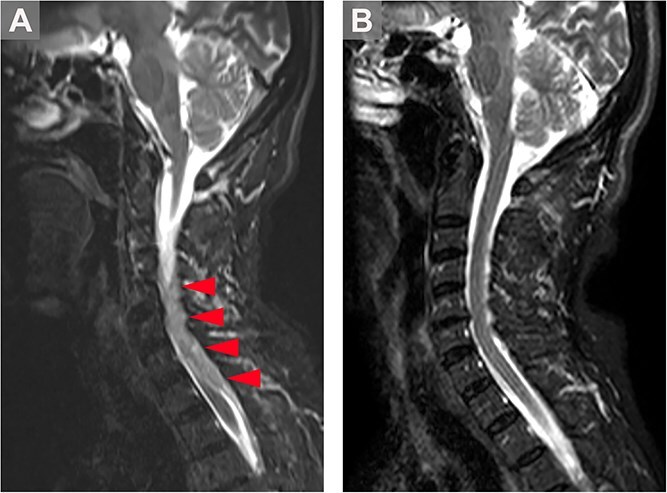
MRI on admission (A, sagittal STIR) reveals a predominantly dorsal cervical epidural hematoma extending from C4 to T2, compressing the spinal cord (arrowheads). Follow-up MRI on day 4 after admission (B, sagittal STIR, long TE) demonstrates a reduction in hematoma size. Abbreviations: STIR, short tau inversion recovery; TE, echo time.

## Discussion

This case illustrates two clinical implications. First, SSEH can present solely with pain, without neurological deficits. Second, it can be detected incidentally on CT performed for other emergencies through systematic evaluation. These findings demonstrate the necessity of clinician awareness and rigorous image interpretation in emergency settings.

Most patients with SSEH present with acute, severe pain followed by neurological deterioration, with approximately 37% developing complete sensorimotor deficits [2]. In contrast, our patient experienced only pain with transient, minimal sensory disturbance. Although uncommon, similar pain-only cases [5] suggest that some patients with unexplained acute spinal pain—including those who recover without imaging—may have undiagnosed SSEH. Early recognition, even without neurological deficits, prevents potential deterioration and provides a definitive diagnosis for cases that might otherwise remain unexplained.

The mechanisms underlying SSEH remain uncertain, which may account for its diverse clinical presentations. The most widely accepted mechanism is rupture of the valveless internal vertebral venous plexus, which is highly susceptible to fluctuations in venous pressure [2]. However, this venous theory may not fully explain cases occurring in the cervical region, where venous pressure is relatively low, prompting some authors to propose an arterial origin [6]. Arterial bleeding typically results in rapid expansion and marked neurological deficits [2, 3]; the absence of these features, combined with spontaneous resolution, supports a venous origin in our patient. Other rare etiologies, such as vascular malformations or underlying coagulopathies, were reasonably excluded based on unremarkable contrast-enhanced CT and laboratory findings.

Given that CT is frequently the initial modality to exclude vascular emergencies, its screening value is significant. While MRI remains the gold standard for diagnosing SSEH [2–4], CT can provide the first clue of SSEH if interpreted with high clinician awareness. A study of 51 SSEH cases revealed that only 16% (8/51) were correctly diagnosed on initial emergency neck CT [7]. Conversely, a single-center descriptive study of 12 patients reported that among the nine who underwent CT, 78% (7/9) had hematomas detectable on CT retrospectively [5]. This disparity suggests that the diagnostic yield of CT depends heavily on the clinician’s awareness. Although CT sensitivity is modest, its high specificity for acute hemorrhage ensures a high positive predictive value once a hyperdense lesion is identified. However, it should be noted that non-contrast CT cannot reliably differentiate hematomas from abscesses or tumors [4]. Therefore, a systematically evaluated CT finding serves as an "early warning" that signals the necessity of an urgent MRI for definitive diagnosis.

The clinical utility of this "early warning" also depends on resource availability. In rural or resource-limited settings where MRI access is unavailable or delayed, recognizing subtle CT findings is vital to expedite transfer to a tertiary facility for definitive MRI and specialized care. Teleradiology can provide expert review in such scenarios, minimizing the potential for diagnostic oversight. Ultimately, such a systematic approach ensures that even patients with resolving symptoms receive a clear diagnosis, preventing these cases from being overlooked in emergency settings.

The management of SSEH depends on neurological severity and progression. While surgical decompression is indicated for patients with severe or worsening deficits, conservative management can be appropriate for those with mild or improving symptoms, with favorable outcomes reported in 73–84% of cases [8–10]. Spontaneous improvement may result from hematoma redistribution within the spinal canal [9]; however, the risk of delayed neurological deterioration requires vigilant clinical monitoring, especially during the first week [10]. The present case demonstrates the feasibility of conservative management in carefully selected patients who remain neurologically stable following the resolution of transient symptoms.

## Conclusion

SSEH should be included in the differential diagnosis of acute posterior neck or back pain, even without neurological deficits. Although MRI remains the gold standard for diagnosis, systematic evaluation of CT soft-tissue windows can provide valuable diagnostic clues, facilitating timely recognition. This initial diagnosis must be followed by close clinical monitoring and follow-up imaging to ensure the safety of conservative management and to confirm hematoma resolution. Maintaining awareness of such atypical presentations is essential to prevent diagnostic oversight and improve patient outcomes.
